# A Tissue-Specific Rhythmic Recruitment Pattern of Leukocyte Subsets

**DOI:** 10.3389/fimmu.2020.00102

**Published:** 2020-02-14

**Authors:** Yinglin Yuan, Shengwang Wu, Weiwei Li, Wenyan He

**Affiliations:** ^1^Medical Center of Hematology, The Xinqiao Hospital of Army Medical University, Chongqing, China; ^2^State Key Laboratory of Trauma, Burn and Combined Injury, Army Medical University, Chongqing, China; ^3^Department of Anesthesiology, The First Affiliated Hospital of Chongqing Medical University, Chongqing, China

**Keywords:** circadian rhythm, leukocyte, recruitment, function, chronopharmacology

## Abstract

The circulating of leukocytes in the vasculature to reach various organs is a crucial step that allows them to perform their function. With a sequence of interaction with the endothelial cells, the leukocytes emigrate from the circulation either by firm attachment to vascular beds or by trafficking into the tissues. Recent findings reveal that the leukocyte recruitment shows time as well as tissue specificity depending on the cell type and homing location. This spatiotemporal distribution of leukocyte subsets is driven by the circadian expression of pro-migratory molecules expressed on the leukocytes and the endothelium. Both the systemic circadian signals and the cell's intrinsic molecule clock contribute to the oscillatory expression of pro-migratory molecules. The rhythmic recruitment of leukocytes plays an important role in the time-dependency of immune responses. It also helps to update blood components and maintain the tissue circadian microenvironment. In this review, we discuss the current knowledge about the mechanisms of the circadian system regulating the leukocyte rhythmic migration, the recruitment pattern of leukocyte subsets into different tissue/organs, and the time-dependent effects behind this process.

## Introduction

Blood leukocyte numbers display circadian rhythms in various mammalian species, like rodents (1) and human (2), with a consistent trend, showing a peak in the resting phase and a trough in the activity phase for most of the leukocyte subsets (3). This process reflects the dynamic emigration of leukocytes from the bone marrow (4) and the recruitment to various organs (3). It has been proved that the major leukocyte subsets, including neutrophils, inflammatory monocytes, non-inflammatory monocytes, CD4 T cells, CD8 T cells, NK cells, and eosinophils, emigrate from the mouse's blood stream and recruit into distinct tissue/organs in a rhythmic manner with the highest homing occurring at the rest-activity transition phase (3). However, the mechanism that governs the tissue-specific rhythmic recruitment pattern and the time-dependent effects brought by this process are not entirely understood.

## The Rhythmic Emigration of Leukocytes is Governed by the Pro-Migratory Molecules

**Figure 1:**
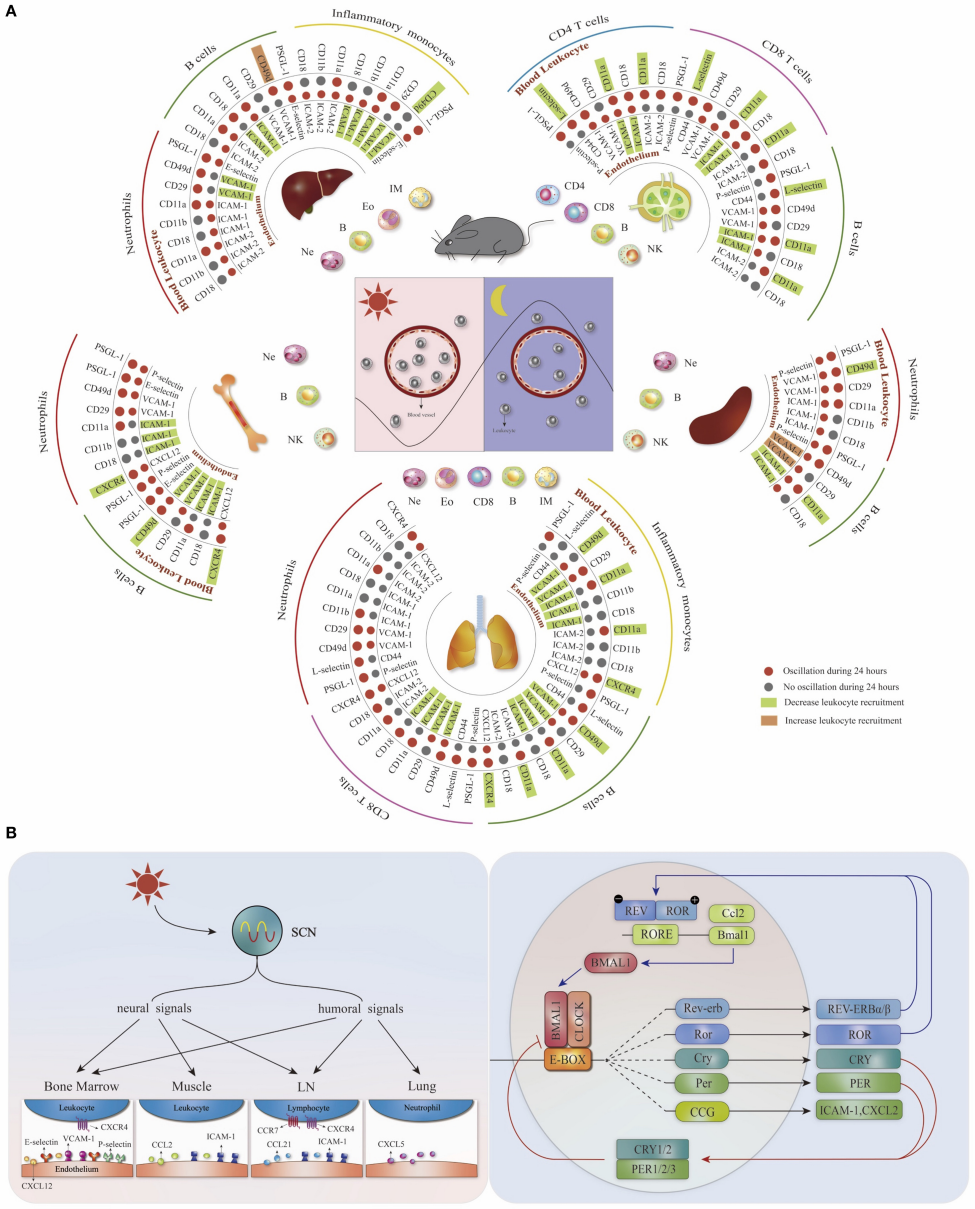
The homing code and the mechanisms for regulating tissue- and leukocyte-specific homing. **(A)** Distinct leukocyte subsets recruited rhythmically (more in the evening) to different organs based on the tissue- and leukocyte- specific oscillatory expression (red dots) of pro-migratory molecules. Targeting different pro-migratory molecules can decrease (green square) or increase (orange square) the homing of specific leukocyte subsets, depending on the cell type as well as homing location. Modified, with permission, from He et al. (3). **(B)** The circadian system regulates the oscillatory expression of pro-migratory molecules through the systemic circadian signals as well as the cell-intrinsic molecular clock.

The leukocyte migration occurs by intensive interaction of adhesion molecules and chemokine receptors with endothelial cells in multiple steps, including adhesion, rolling, crawling, and transmigration (5). Through a time-based screening approach, the expression oscillations of adhesion molecules and chemokine receptors on the surface of different leukocytes and vascular beds in mice have been identified (3, 6) (Figure 1A). Blocking receptor–ligand interactions on the endothelium (ICAM-1 or VCAM-1) or the leukocytes (CXCR4, L-selectin, CD11a, or CD49d) abolishes the rhythmicity of leukocyte emigration from murine blood, suggesting that both leukocytes and the microenvironment contribute to the time-dependent homing process (3). Moreover, the optimal efficacy of those migration blockers is acquired at the rest-activity transition time point when ICAM-1, VCAM-1, CXCR4, and CD49d reach their peak expression levels (3), indicating that the circadian recruitment of leukocytes is dependent on the oscillatory expression of pro-migratory molecules. In addition to this time-dependent feature, the expression of pro-migratory molecules exhibits leukocyte and tissue specificity. Different mouse leukocyte subsets display a rhythmic homing profile to distinct organs, which can be inhibited by targeting specific pro-migratory molecules (Figure 1A) (3). Consequently, these data implicate that the pro-migratory molecules with time- and tissue- specific expression signatures form a homing code that guides the homing of different leukocyte subsets.

## The Circadian System Regulates Leukocyte Rhythmic Homing

The circadian rhythm is self-sustained, which is primarily attributed to the autonomous molecular clock consisting of a transcription–translation feedback loop. Briefly, two central transcription factors, CLOCK (circadian locomotor output cycles kaput) and BMAL1 (brain and muscle Arntl-like protein 1, also known as ARNTL), form a heterodimer and bind to Enhancer Boxes (E-box) containing sequences to induce the expression of clock-controlled genes, including their negative regulator *Per1/2/3* (period circadian protein homologs) and *Cry1/2* (cryptochromes), which inhibit further transcriptional activation by CLOCK/BMAL1 complex. An accessory feedback loop involves REV-ERBα and REV-ERBβ proteins (encoded by *Nr1d1* and *Nr1d2*), which compete with ROR proteins to inhibit *Bmal1* transcription via Rev-Erb/ROR response elements (RORE) (7, 8). The circadian genes can modulate leukocyte migration by regulating pro-migratory molecules. *Bmal1* is the major target for diminishing circadian rhythms as the single knockout induces molecular and behavioral arrhythmicity in mouse (8). B-cell (*CD19-cre*) and neutrophil (*Lyz2-cre*)-specific *Bmal1* deletion abolish the time-of-day differences in the expression of CD11a on B cells and PSGL-1 on neutrophils, thus ablating their homing rhythmicity to the spleen (3). Endothelial cell-specific *Bmal1* knockout (*Cdh5–cre*ERT2: *Arntl flox*) mice lose the circadian expression of VCAM-1 and ICAM-1 on the endothelium of the lung and liver, respectively, resulting in the arrhythmic homing of leukocytes to these two organs (3). With chromatin immunoprecipitation assays, BMAL1 has been found to bind to the neutrophil E-box element of *Cxcl2* to increase its expression, which induces neutrophil aging (9) that have higher expression levels of CD11b and CD49d (10). CLOCK binds to an E-box-like enhancer of *Icam-1* and regulates the adhesion of mononuclear cells to endothelial cells by increasing the expression of ICAM-1 and adhesion related genes on the cultured endothelial cells (11). In addition to BMAL1 and CLOCK, other proteins involved in the molecule clock loop also play a role in regulating the expression of pro-migratory molecules. Rev-erbα binds to the RORE of *Ccl2* to repress its expression in mouse macrophages and impairs cell adhesion and migration (12). Overexpression of *Cry1* reverses the increased VCAM-1, ICAM-1, and E-selectin expression on the vascular endothelial cells in sleep deprivation mice and suppresses the binding of monocytes to endothelial cells (13). These studies further support that the circadian clock genes regulate the oscillatory expression of pro-migratory molecules and modulate leukocytes migration.

The peripheral clocks are synchronized by the suprachiasmatic nucleus (SCN) located in the hypothalamus to be phase coherent with the environment. The environment light conditions are transferred into photic neural input by the eye and transmitted to the SCN central clock via the retinohypothalamic tract (14). This timing information is further transmitted from the SCN to the peripheral clocks through two major pathways, neural and humoral signals (8); both can influence the rhythmic recruitment process of leukocytes through regulating pro-migratory molecules. The sympathetic nerves act on a β-adrenoreceptor to synchronize the emigration of mouse leukocytes to the bone marrow and muscles by inducing tissue-specific oscillation of endothelial cell adhesion molecules and chemokines (P-selectin, E-selectin, and VCAM-1 in the bone marrow as well as ICAM-1 and CCL2 expression in the skeletal muscle) (6). The increased nocturnal homing of mouse leukocytes to the bone marrow is mediated by increased expression of E-selectin and VCAM-1 on the endothelium, which are controlled by the interplay between cholinergic signals and sympathetic noradrenergic tone (15). In terms of the hormone pathway, higher cortisol level in the morning increases the CXCR4 expression of human CD4 T cells and may guide them to the bone marrow (16). The noradrenaline secreted by the sympathetic nerve regulates the oscillatory CXCL12 expression in the bone marrow and elicits the egress of hematopoietic stem cells in a circadian manner (4). In addition, corticosterone oscillations regulate rhythmic murine CXCL12 and CXCR4 expression by bone marrow stromal progenitors (17). These observations demonstrate that systemic circadian signals can drive the oscillatory expression of pro-migratory factors.

The mechanism behind how the systemic circadian signals regulating the expression of pro-migratory molecules through the Bmal1 dominated molecular clock remains elusive. With a humanized mouse model, Zhao et al. have proved that the inner circadian environmental change is connected with the cell molecule clock through the p38 mitogen-activated protein kinases/mitogen-activated 2 (MAPK/MK2)-ROS-HIF-1a-ARNTL1 pathway, which results in an opposite ROS level and inverse oscillation trend of CXCR4 expression in human and mouse leukocytes (18). Rhythmic glucocorticoids regulate the circadian expression of CXCL5 by mouse lung epithelial club (Clara) cells. Genetic ablation of Bmal1 in bronchiolar cells disrupts the CXCL5 rhythms despite persistent oscillatory glucocorticoid levels (19). Collectively, these studies suggest that the clock genes link systemic circadian signals with the oscillatory expression of pro-migratory molecules.

Together, these findings indicate that the circadian system regulates the oscillatory expression of pro-migratory molecules, thus influencing the homing process. Both the cell autonomous clock and the rhythmic microenvironment play an important part in the rhythmic homing of leukocytes into various organs (summarized in Figure 1B).

## Tissue-Specific Homing of Leukocyte Subsets and the Time-Dependent Effects

Leukocyte subsets migrate to different tissues with the help of the ligand–receptor interaction between the leukocytes and the endothelium. In this part, we summarize the driving molecules for the rhythmic homing of leukocyte subsets to specific tissue/organs and the functional role behind the rhythmic homing behavior (Table 1).

**Table 1:** Tissue specific leukocyte rhythmic homing and their effects.

**Organ**	**Rhythmic homing cells**	**Driving molecules**		**Conditions**	**Effects**	**Reference**
		**Leukocyte**	**Vascular bed**			
Lymph node	CD4 T cells, CD8 T cells	CCR7	CCL21	Steady state /EAE/Helicobacter pylori/influenza A virus	Heightened acquired immune function when stimulus occurred while lymphocytes accumulated in the lymph nodes	(20)
	CD4 T cells, CD8 T cells	CXCR4		Steady state	More rapid proliferation and efficient migration of lymph node T cells at night	(21)
Bone marrow	Neutrophils	CXCR4	CXCL12	Steady state	Neutrophil clearance modulates the hematopoietic niche, which contributes to the rhythmic egress of hematopoietic progenitors	(22)
Lung	Neutrophils			Steady state	Neutrophil aging and apoptosis	(3, 23)
			CXCL5	Inflammation	Time of day variation in the pulmonary inflammation and responses to bacterial infection	(24)
	B cells	CXCR4, CD11a, and CD49d	VCAM-1, ICAM-1	Steady state	Neutrophil aging and apoptosis	(3, 23)
Heart	Neutrophils	CXCR2	VCAM-1, ICAM-1, CXCL1, CXCL2, CXCL5, CCL3, and CCL5	Myocardial infarction	MI at ZT13 induces enhanced neutrophil infiltration and leads to poor prognosis	(25)
	Monocytes	CCR2	CCL2	Myocardial infarction	MI at ZT13 induces enhanced monocytes infiltration	(26)
Vessel	Neutrophils and monocytes	CCR2	CCL2	Atherosclerosis	Timed regime of blocking CCR2 during the activity phase inhibits atherosclerosis	(27)
	Neutrophils	CD11a, CD11b, CCR2 (artery) CD11a, CD11b, CCR2, CXCR2 (vein)	ICAM-1, VCAM-1	TNF-α induced acute inflammation	Time shifted leukocyte recruitment between artery and vein results in different thrombus formation time	(28)

### Lymph Node

The lymph node(LN) is mainly composed of CD4 T cells, CD8 T cells, and B cells, which are also the major leukocyte populations that home to the mouse lymph node (3). The lymphocytes accumulate in the mouse lymph node at the beginning of the night due to increased homing and reduced migration at this time point (20), which is governed by the dynamic expression of pro-migratory molecules including chemokine receptor CCR7, ICAM-1, and CCL21 on the high endothelial cell venules (3, 20). T-cell specific (*CD4-cre*) *Bmal1* knockout diminishes the rhythmic expression of CCR7 and thus ablates the rhythmic homing process of T cells (20), suggesting that the rhythmic recruitment of lymphocytes to LN is determined by the oscillatory expression of specific pro-migratory molecules mediated by the circadian clock. In addition, the sympathetic nerve regulates the rhythmic homing of lymphocytes to LN through β_2_ adrenergic receptors (29), but it is unclear what pro-migratory molecules are controlled by the neural signaling in this process. The glucocorticoid receptor signaling elevates CXCR4 expression, which redistribute T cells between lymphoid organs and blood (21). These findings demonstrate that the rhythmic lymphocyte recruitment to LN is controlled by multiple circadian factors, including the molecule clock as well as the neural and humoral signals.

This diurnal oscillation of leukocyte number contributes to the time-dependent humoral immune response. Immunization of mice during the period of lymphocyte accumulation in LNs increases antibody titers (29). The severity of the experimental autoimmune encephalomyelitis mouse model is dependent on the time point of immune stimulation, and *CD4-cre Bmal1* knockout mice lose this time-dependent difference (20), suggesting that T cell rhythmic migration affects the time-dependent immune reaction. The influence of the LN rhythms is further demonstrated by immune response to pathogens. Mice infected with influenza A virus at ZT8 (ZT, *zeitgeber time*, ZT0 refers to light onset) can lead to stronger extent of pulmonary CD8^+^IFN-γ^+^ T-cell infiltration than at ZT20, 8 days post infection (20). Together, these data strongly indicate that the adaptive immune responses follow a circadian rhythm according to the LN number change.

In addition to lymphocytes, NK cells also exhibited a strong homing rhythmicity to the LN (3). Homed NK cells reside in the paracortex and the medulla of LN where they can be in contact with dendritic cells (DC). In addition, NK cells regulate colocalized T-cell responses in L. major infections through secreting INF-γ (30), but whether this NK–DC–T-cell interaction contributes to the time-dependent immune function needs to be further explored.

### Bone Marrow

The bone marrow produces and releases leukocytes for the blood replenishment in a rhythmic manner, which is entrained by the sympathetic nerves (4). In addition, it's also an important site for mouse leukocyte rhythmic homing, including neutrophils, B cells, and NK cells (3, 22). The homing of mouse neutrophils to the bone marrow is dependent on CXCR4, ICAM-1, and L-selectin (3). Due to a short life span, the aged mouse neutrophils that shed L-selectin and exhibit high CXCR4 expression are dynamically eliminated from the circulation, which can clear the aged neutrophils and keep the immune homeostasis (22). Bone marrow macrophages engulf aged neutrophils and modulate the niche environment, resulting in the releasing of progenitor cells (22). Therefore, the rhythmic neutrophil homing links the blood environmental change and the bone marrow regeneration capacity.

In addition to neutrophils, B cells and NK cells also migrate into the mouse bone marrow in a circadian manner. B-cell homing to the bone marrow depends on VCAM-1, ICAM-1, CXCR4, and CD49d (3). The homed B cells reside in the perisinusoidal niche of the bone marrow and can freely recirculate and respond to blood-borne microbes, which extends the function of bone marrow as a secondary lymphoid organ (31). NK cells home to the bone marrow during viral infection for the preservation of their ability to respond to subsequent viral challenge (22). These studies demonstrated that the recruitment of B cells and NK cells to the bone marrow is closely related to the immune responses. Therefore, the rhythmic migration behavior of those cells may influence the immune function in a time-dependent way.

### Lung

The lung vasculature has a continuous and non-fenestrated endothelium to facilitate gas exchange and perform barrier functions (32). Under this special morphology, leukocytes recruit to the lung by firm attachment to the vessel wall, temporarily sequestered from the circulation blood, making the lung a reservoir of leukocytes (33).

In the steady state, mouse neutrophils home to the lung in a rhythmic manner, with more neutrophils attached to the vasculature at night (3, 34). Unlike the lymph node, spleen, and bone marrow, the homing of neutrophils to the lung is L-selectin independent (3), and some studies have suggested that spatial constrains seem to dominate neutrophil retention in lung capillaries (35). The diurnal infiltration of neutrophils to the lung maintains the rhythmic microenvironment of this organ, which controls the melanoma metastasis rhythm. Neutrophil-specific (*Lyz2-cre*) Bmal1 deletion abolishes this rhythmic recruitment of neutrophils as well as the melanoma metastasis rhythm (34), suggesting that the diurnal microenvironment of the lung is regulated by rhythmic neutrophil infiltrations and can change the susceptibility to diseases. For neutrophils, the lung provides a niche for the recruited neutrophils to encounter with B cells, which transfer MHCII to neutrophils and induce neutrophil aging (23). These data highlight that the rhythmic leukocyte homing is crucial for both the leukocyte physiology and the tissue circadian environment.

The homing of mouse neutrophils to the lung is also rhythmic in LPS-triggered pulmonary inflammation with more neutrophil infiltration in the daytime, which is induced by rhythmic expression of CXCL5 secreted by lung Clara cell (24). Although Clara cells have been shown to be under direct control of the glucocorticoid receptor-medicated repression, the neutrophilia persists with glucocorticoid receptor-depleted mice, suggesting other factors might be involved in neutrophil rhythmic migration in the inflammatory scenario (19). These data suggest that the circadian control of pro-migratory factor is interplayed between multiple factors.

Besides neutrophils, mouse B cells, CD4 T cells, and inflammatory monocytes also recruit rhythmically to the lung. Blocking VCAM-1, ICAM-1, CD11a, or CD49d decreases the homing of these two cells (3). Future studies are needed to investigate the functions of the rhythmic homing of B cell and CD4 T cells to the lung and the interaction between different leukocyte subsets during their migration.

### Heart and Vessel

The acute ischemic vascular events exhibit strong time-of-day dependency. Myocardial infarction (MI) occurs predominately in the morning in humans and is associated with unfavorable outcomes (36–38). This finding is well-reproduced in mouse MI model by permanent ligation of the left anterior descending coronary artery. Mice subjected to the MI model at ZT13 result in higher cardiac infarct size with more infiltration of neutrophils and monocytes compared to ZT5 (25, 26). At steady state, neutrophils and monocytes home rhythmically to the heart with the highest infiltration occurred at ZT13 during 24 h, and this time-dependent difference is greatly enhanced in MI situations depending on higher expression of CXCR2 and CCR2 on neutrophil and monocytes, respectively, together with corresponding adhesion molecules and chemokines on the endothelium (25, 26). Targeting CCR2 significantly inhibits monocyte infiltration at ZT13 but not ZT5 in the case of MI (26). Inhibiting the chemokine receptor CXCR2 or using neutrophil-specific CXCR2 knockout remarkably reduces the infarct size and preserves cardiac function after the occurrence of MI (25). These data suggest that the chrono-pharmacological treatment targeting the rhythmic migration behavior of the leukocytes can provide a favorable treatment outcome. The influence of the circadian system on MI is further demonstrated by the homozygous clock mutant mice(*Clock*^delta19^), which leads to more infiltrations of neutrophils and macrophages to infarcted myocardium, and worse cardiac structure, suggesting that abolishment of circadian rhythm can adversely affected the cardiac remodeling (39). REV-ERB agonist SR9009 provides beneficial effects in long-term cardiac repair for mice post-myocardial ischemia reperfusion (40). Collectively, the circadian rhythm modulating inflammatory responses is crucial for the myocardia recovery and prognosis.

The atherosclerosis is a chronic inflammation of the arterial wall, which greatly involves leukocyte recruitment and can lead to acute cardiovascular events (41). Myeloid cells recruit to the atherosclerotic lesions in a circadian fashion with a peak during the early activity phase, which is driven by rhythmic level of myeloid cell-derived CCL2 immobilized on the endothelial cells. CCR2 on myeloid cells shows in phase oscillation with CCL2. Together, CCL2-CCR2 triggers rhythmic adhesion of myeloid cells. Myeloid *Bmal1*-specific knockout mice (*Lyz2-cre*: *Arntl flox*) lose rhythmic expression of CCL2 and oscillation of myeloid cell adhesion, suggesting that the circadian expression of CCL2 mediated by BMAL1 is responsible for the rhythmic homing of myeloid cells. The rhythmic behavior of myeloid cells provides a timed treatment strategy for CCR2 neutralization with antagonist RS102895, which efficiently reduced atherosclerosis with a short-time administration at ZT17 without disturbing microvascular recruitment (27), supporting the concept that the time and site-specific leukocyte recruitment pattern provides us with a beneficial manner to regulate the leukocyte migration with time-tailored therapy.

Besides atherosclerosis, thrombotic vascular occlusion also exhibits strong circadian rhythm, which is strongly dependent on the circadian clock control (42). A recent study reveals that myeloid cells recruit to arteries and veins in a phased delayed rhythmic manner in the TNF-α induced acute inflammation model. This time shift of myeloid cell adhesion is regulated by a vessel type specific oscillatory pattern in the expression of pro-migratory molecules, driven by the intrinsic autonomous clock as well as local sympathetic innervation. The distinct leukocyte adhesion patterns in the vein and artery result in a different acute thrombus formation time in the vein (at ZT2) and artery (at ZT8) in a phototoxicity-induced thrombus model (28). The experiments described above highlight the different circadian rhythmicity between veins and arteries.

## Conclusion

In summary, leukocyte subsets home to different tissue/organs governed by the interaction of distinct pro-migratory molecules expressed on endothelial cells and leukocytes. The circadian system regulates the time-dependent expression profiles of the pro-migratory molecules by multiple factors that are only partially known. Future studies are needed to investigate how the synchronization signals interplay with the peripheral molecule clock to regulate the expression of pro-migratory molecules. The leukocyte rhythmic homing plays important functional roles in various aspects, including immune responses, the replenishment of the blood, as well as the tumor metastasis rhythms. Targeting specific leukocyte rhythmic homing in a time-tailored way has proven to be a beneficial method in some pathological conditions. To create new possibilities for the chronotherapies for human patients, more experiments with mechanistic insights into the circadian leukocyte migration are needed. In addition, the functional roles of leukocyte rhythmic trafficking should be further explored.

## Author Contributions

YY and SW drafted the article and designed the figure. WH devised the structure of the review and wrote the manuscript. WL aided in revising the manuscript. All authors give approval for publication.

### Conflict of Interest

The authors declare that the research was conducted in the absence of any commercial or financial relationships that could be construed as a potential conflict of interest.
